# Association Between Severity of COVID-19 Infection and Persistent Dyspnea in Recovered Adults: A Cross-Sectional Study

**DOI:** 10.7759/cureus.85666

**Published:** 2025-06-09

**Authors:** Atif Saeed, Marium Nadeem Khan, Momina Kamran, Shivam Singla, Sri Teja Chikkala, Moeed Akbar Malik, Joseph Benjamin Baidoo, Farhan Ullah, Bhavna Singla, Sara Ali, Fatima Alam

**Affiliations:** 1 Emergency Medicine, Ungoofaaru Regional Hospital, Ungoofaaru, MDV; 2 Medicine, Shifa College of Medicine, Islamabad, PAK; 3 Medicine/Internal Medicine, Allama Iqbal Medical College, Lahore, PAK; 4 Internal Medicine, Hackensack Meridian Ocean Medical Center, Brick, USA; 5 General Internal Medicine, Northern Care Alliance, Royal Oldham Hospital, Greater Manchester , GBR; 6 Medicine, Shaheena Jamil Teaching Hospital, Abbottabad, PAK; 7 Internal Medicine, North Sichuan Medical College, Nanchong, CHN; 8 Internal Medicine, Khyber Teaching Hospital, Peshawar, PAK; 9 Internal Medicine, Erie County Medical Center (ECMC) Hospital, Buffalo, USA; 10 Internal Medicine, Gujranwala Medical College Teaching Hospital, Gujranwala , PAK; 11 Medicine, Lumina Research Foundation, Islamabad, PAK

**Keywords:** covid-19, covid-19 severity, dyspnea severity, long-term effects, persistent dyspnea

## Abstract

Introduction

The COVID-19 pandemic has led to a high number of survivors with persistent symptoms, such as dyspnea, long after recovery. It is important to understand the association between the severity of the initial COVID-19 infection and the persistence of dyspnea to guide patient management and rehabilitation planning.

Methods

This cross-sectional analysis involved adult patients who had recovered from laboratory-confirmed COVID-19 infection. Patients were stratified into groups by the severity of their acute infection (mild, moderate, severe) based on the WHO Clinical Progression Scale. Persistent dyspnea was measured with a validated dyspnea scale. Statistical analysis was conducted to examine the association between COVID-19 severity and severity of dyspnea, adjusting for potential confounders.

Results

A total of 385 individuals took part in the study, where 217 people (56%) fell within the 30 to 39 years age bracket, while female participants constituted 52% (200 individuals). The respondents were primarily widowed people who had received secondary education (n = 119, 31%, and n = 118, 31% respectively). A total of 141 participants were current smokers, while 145 participants reported never smoking. Among the study participants, 73 experienced asthma (19%), and hypertension affected 68 individuals (18%). The COVID-19 severity of patients spanned from mild (n = 144, 37%) to critical (n = 78, 20%), and 51% of patients received hospital care (n = 197). Research findings indicated that dyspnea symptoms and advancement in the disease are closely linked (r = 0.521, p < 0.05). People who got oxygen therapy had higher clinical scores than those who did not (p = 0.01). When comparing the ICU patients to other patients, no important differences were noticed (p = 0.05). Recovery was felt very minimally in the results. According to regression analysis, changes in the patient’s clinical condition were highly associated with dyspnea, as shown by a β value of 0.521 and p < 0.001.

Conclusion

The intensity of COVID-19 infection strongly correlates with both the enduring nature and severity of dyspnea in adults who have recovered from the disease. Post-COVID follow-up and rehabilitation programs require strong evidence behind them to support patients who have experienced severe COVID-19 infections.

## Introduction

Humanity has endured countless epidemics across history, with COVID-19 ranking among the most widely spread globally in recent times [1]. COVID-19, resulting from SARS-CoV-2 and first appearing in Wuhan in December of 2019, created a worldwide pandemic with significant health and societal consequences. Most recover, but many, particularly those who are moderately or severely ill, have persistent symptoms such as dyspnea [2-4].

The host immune response is central to COVID-19 pathogenesis, with severe infections typically characterized by excessive inflammation and immune dysfunction. Their understanding and control are prerequisites for enhancing clinical outcomes and informing practical treatment approaches [5]. COVID-19 has resulted in almost 200 million cases and four million deaths, with vaccine development showing remarkable progress. As of mid-2021, three billion doses were distributed, highlighting the requirement for worldwide access to vaccines to put an end to the pandemic [6]. COVID-19 has dramatically affected everyday life and decelerated the global economy, triggering widespread sickness and mortality [7]. Neurological complications have now emerged as a cause for concern during the COVID-19 pandemic, with symptoms varying from headache and loss of smell to confusion and stroke [8].

Chronic dyspnea, which is a frequent post-COVID-19 condition, occurs in 26% to 41% of patients, and risk factors are female sex, high BMI, pulmonary comorbidities, and severity of COVID-19 illness [9]. Chronic dyspnea is frequently described in those with prolonged COVID, even when cardiopulmonary impairment is not detectable [10].

Post-COVID-19 patients with chronic dyspnea exhibit decreased functional capacity, increased inflammatory markers, and altered left atrial strain. These results indicate that cardiac dysfunction and systemic inflammation can contribute to long-term respiratory symptoms [11]. Over one-third of the recovered COVID-19 patients had persistent dyspnea after one year, even with normal ejection fraction. These residual symptoms may be caused by subclinical cardiac dysfunction, indicated by decreased myocardial work indices (GCW and GWI) [12].

Fatigue and dyspnea are the most common long-term symptoms after COVID-19, persisting in many patients for up to a year [13]. Prolonged COVID-19 severity is associated with chronic dyspnea and fatigue, but not always with other long-term symptoms. All survivors of COVID-19, irrespective of initial severity of illness, can have lingering effects and need continued symptom surveillance and monitoring [14].

The research aims to find a relationship between COVID-19 severity of infection and the persistence of post-COVID dyspnea among recovered adult patients. Recognizing the relation might help practitioners learn more about complications following COVID-19 infection and inform management programs in the long term for afflicted patients.

Rationale

While several studies have investigated the association between the severity of COVID-19 illness and residual symptoms, including persistent dyspnea, many have emphasized only hospitalized or ICU patients and did not have thorough stratification by severity categories. Furthermore, variability in symptom measures hinders comparisons between studies and reduces the generalizability of results. This research takes a novel approach by having a vast, community-based population of recovered adults, ranging from mild to severe baseline infections. In contrast to previous studies that tend to exclude non-hospitalized patients or are short-sighted on long-term respiratory impacts in this subgroup, this research considers them as well, even if they might not have been formally seen post-COVID. In addition, it delves into how demographic and clinical characteristics like sex, age, and comorbidities can potentially affect the relationship between the severity of initial COVID-19 and persistent dyspnea, providing a more refined and comprehensive explanation for post-COVID respiratory consequences.

Objectives

This research aims to: (i) investigate whether the severity of initial COVID-19 infection (mild, moderate, severe) is associated with the persistence of dyspnea in recovered adults. The primary question of research is whether more severe COVID-19 infections lead to longer-lasting or more severe dyspnea; (ii) assess the prevalence and severity of persistent dyspnea among adults recovering from COVID-19 using the Dyspnea-12 score as the primary outcome measure; (iii) explore how demographic factors (like age and gender) and existing health issues (such as obesity, hypertension, and diabetes) play a role in the development and intensity of chronic dyspnea; (iv) determine if non-hospitalized COVID-19 patients experience chronic dyspnea and identify the factors that contribute to this condition; (v) compare the levels of dyspnea between different groups based on the severity of their COVID-19 experience (like ICU patients versus those who weren’t in the ICU) and see if there are any differences linked to the type of medical care they received during the acute phase of the infection; (vi) offer insights into the long-term respiratory effects for patients who went through varying degrees of COVID-19 infection, which will help us better understand the health complications that can linger after COVID-19.

## Materials and methods

Study design and methods

This study employed a cross-sectional design to investigate the relationship between COVID-19 infection severity and the persistence of dyspnea in recovered adults. The study involved a community-based sample of COVID-19-recovered patients, including both hospitalized and non-hospitalized patients with varying severities of infection.

The study was conducted in Islamabad, Pakistan, utilizing recruitment from outpatient post-COVID care clinics and primary healthcare centers. These offered a representative sample of recovered adults, consistent with the overall population of individuals recovering from COVID-19. Clinical and demographic assessment of factors determining persistent dyspnea was facilitated by research design, making an important contribution to the description of post-COVID respiratory consequences.

Sample size and technique

The study used an infinite population formula to calculate the sample size since the overall population was unknown. The formula is given as:

Sample size = Z² × p(1-p) / d²

Where Z is the statistical value at the desired level of confidence, p is the expected proportion or prevalence, and d is the margin of error or precision. For a 95% confidence level, the value of Z is 1.96, and the margin of error is fixed at 0.05. The assumed prevalence was obtained from a similar study in Pakistan with almost the same participants, which had a prevalence of 40.7%. Thus, p is fixed at 0.407. With these values applied, the estimated sample size is 385 [15].

Study participants were recruited through a convenience sample process at outpatient post-COVID care clinics, together with primary healthcare centers distributed throughout Islamabad. By using this method, the study participant selection process ensured representation from diverse demographic backgrounds with different COVID-19 experience ranges from home recovery to hospitalization admission. To participate in the study, the participants needed to satisfy the following criteria: (i) Adults aged 18 years or older, (ii) With RT-PCR or antigen confirmed COVID-19 infection history, (iii) Recovered from COVID-19 infection at the time of enrollment, and (iv) The participants needed to consent voluntarily to the study. At the same time, the exclusion criteria included both pre-existing respiratory diseases, such as asthma and interstitial lung disease, and known heart conditions that caused breathing difficulties, and unavailability of complete medical records or participant refusal. The sampling technique, together with detailed inclusion and exclusion criteria, made sure the study population mirrored the adult recovery population without biases.

Data collection tools and procedure

The research data collection instrument included three main sections of demographic data and two established rating scales. The demographic portion enabled researchers to document participant details, which included age, occupation, gender, and comorbidity diagnoses such as hypertension and diabetes, and obesity, since these variables influence post-COVID respiratory outcomes.

The initial COVID-19 severity assessment relied on the World Health Organization (WHO) Clinical Progression Scale to place subjects into standardized 10-point categories, which defined their medical condition based on their presentation symptoms and received treatments. The severity of COVID-19 infection determines placement on a scale that runs from no infection (0) to fatal outcomes (10). The study analyzed participants through three distinct groups according to their score results: mild (1-3) and moderate (4-5), and severe (6-9). The scale was established by the WHO Working Group on the Clinical Characterization and Management of COVID-19 infection in 2020 [16].

The Dyspnea-12 scale provided persistent dyspnea measurement by assessing physical and emotional aspects through 12 items that were rated from 0 to 3 (severe). The total score was from 0 to 36. The research tool designed by Yorke et al. in 2010 allowed comprehensive dyspnea evaluation regarding intensity and impact post-COVID infection. This tool demonstrated excellent internal reliability (Cronbach’s alpha = 0.9) [17].

The data collection process occurred via trained staff who performed interviews with patients at their outpatient and primary healthcare clinics. All participating subjects granted their informed consent before study participation began.

Statistical analysis

IBM SPSS Statistics for Windows, Version 26 (Released 2019; IBM Corp., Armonk, New York, United States) was used to enter and analyze the data. The study employed both inferential and descriptive statistical procedures. Demographic and clinical information were summarized through descriptive statistics. Inferential procedures involved Pearson correlation to establish relations among clinical measures, independent t-tests and one-way ANOVA for a comparison of means between the groups, multiple regression to predict the dyspnea, and chi-square for analyzing relationships among categorical data such as chronic illness, number of days at hospital, COVID-19 severity, and length of recovery. Through these, in conjunction, complete data analysis was carried out. The established threshold for statistical significance used a p-value of less than 0.05.

Ethical considerations

The Institutional Review Board (IRB) of Lumina Research Foundation, Islamabad, Pakistan, issued Ethical approval through IRB-2024-0093. Before participants entered the study, they received information about its purpose, followed by written consent. The research projects operated voluntarily so that participants could leave without risk at any time. Throughout the research, the data remained confidential, and a strict adherence to privacy and ethical procedures maintained the use of personal information exclusively for research purposes. Data was collected from January 2025 to April 2025.

## Results

Table 1 demonstrates that 385 people became part of the research. The study participants consisted primarily of individuals within the 30-39 age group (N = 217, 56%), seconded by participants in the 40-49 age group (N = 143, 37%) then followed by those aged 50-59 years (N = 20, 5%) and finally those under 18-29 years old (N = 5, 1%). The collective group consisted of 200 female participants (52%), together with 177 male participants (46%) and eight participants who did not want to reveal their gender identity (2%).

**Table 1:** Demographic characteristics of participants (N=385) f: frequency; %: percentage

Variable	f	%
Age		
18-29 years	5	1
30-39 years	217	56
40-49 years	143	37
50-59 years	20	5
Gender	-	-
Male	177	46
Female	200	52
Prefer not to say	8	2
Marital status		
Single	89	23
Married	93	24
Widowed	119	31
Divorced	84	22
Educational level		
No formal education	35	9
Primary	59	15
Secondary	118	31
College/university	90	23
Postgraduate	83	22
Employment status		
Student	66	17
Employed	109	28
Unemployed	101	26
Retired	70	18
Other	39	10
Smoking status		
Current smoker	141	37
Former smoker	99	26
Never smoked	145	38
History of Chronic Illness	-	-
Hypertension	68	18
Diabetes	61	16
Asthma	73	19
COPD	72	19
Heart disease	64	17
Kidney disease	47	12
COVID-19 Severity	-	-
Mild (no pneumonia)	144	37
Moderate (pneumonia, no oxygen)	72	19
Severe (oxygen required)	91	24
Critical (ICU/mechanical ventilation)	78	20
Hospitalized for COVID-19	-	-
Yes	197	51
No	188	49
Duration of hospitalization	-	-
Less than one week	105	27
1-2 weeks	122	32
More than two weeks	158	41
Received oxygen therapy during infection	-	-
Yes	249	65
No	136	35
Received ICU care	-	-
Yes	175	45
No	210	55
Time since recovery	-	-
Less than one month	80	21
1-3 months	219	57
4-6 months	74	19
More than six months	12	3

The survey results indicated that widowed participants (N = 119, 31%) formed the largest marital group, while married respondents (N = 93, 24%) and single participants (N = 89, 23%) came next, followed by those who divorced (N = 84, 22%). The participants who had finished secondary education represented the largest group (N = 118 and 31% of the total) yet college or university graduates also formed a significant portion (N = 90 and 23% of total participants) and postgraduate (N = 83 and 22% of total participants), primary education (N = 59 and 15% of total participants) and without any formal education (N = 35 and 9% of total participants).

The analysis revealed that 109 workers represented 28% of the sample, while 101 participants were unemployed at 26% and 70 retirees made up 18%, students amounted to 17%, and 39 participants selected other types of employment at 10%. Among the respondents, 145 participants (38%) declared they had never smoked, yet 141 (37%) persisted in smoking, while 99 (26%) reported being former smokers. The study revealed that 73 patients (19%) experienced asthma while 72 (19%) had COPD and 68 patients (18%) had hypertension, and 64 had heart disease (17%), among others. The participants also reported having diabetes (16%), while 47 patients (12%) had kidney disease. The study grouped COVID-19 infection levels into four categories, where 37% of participants had mild illnesses, 19% had moderate conditions, and 24% experienced severe COVID-19, while 20% fell under the critical category.

Hospitalization for COVID-19 affected more than half of the study participants (N = 197, 51%), yet 188 participants (49%) did not need hospital treatment. Hospitalization lengths differed among patients, with 105 participants (27%) spending under one week in hospital, followed by 122 participants (32%) who stayed 1-2 weeks, and 158 participants (41%) who needed hospitalization longer than two weeks. The study results indicated that oxygen therapy was administered to 249 participants among 380 patients, while the number receiving ICU care reached 175 patients. Many participants (N = 219, 57%) experienced recovery between 1 and 3 months ago while 80 (21%) recovered less than one month earlier and 74 (19%) recovered during the period of 4 to 6 months ago and 12 (3%) had not reached their complete recovery state after six months.

Table 2 shows the findings of two normality tests applied to the Ordinal Clinical Progression Scale and the Dyspnea-12 Questionnaire scores using Kolmogorov-Smirnov and Shapiro-Wilk tests. Both variables gave statistically significant results (p < 0.001), revealing that their distributions are quite far from normal. The results for the Ordinal Clinical Progression Scale were highly skewed, with a Kolmogorov-Smirnov statistic at 0.362 and a Shapiro-Wilk statistic at 0.686, and also, the Dyspnea-12 Questionnaire demonstrated a deviation from a normal distribution (Kolmogorov-Smirnov statistic = 0.160 and Shapiro-Wilk statistic = 0.932). Because the assumptions for normality are not met, it is better to use non-parametric statistical methods for further analysis of these variables.

**Table 2:** Assessment of data normality for Ordinal Clinical Progression Scale and Dyspnea-12 Questionnaire using Kolmogorov-Smirnov and Shapiro-Wilk tests df: degree of freedom; *: p<0.05, **: p<0.001 considered significant

Variables	Kolmogorov-Smirnov			Shapiro-Wilk		
	Statistic	df	p	Statistic	df	p
Ordinal Clinical Progression Scale	0.362	385	<0.001	0.686	385	<0.001
Dyspnea-12 Questionnaire	0.160	385	<0.001	0.932	385	<0.001

Table 3 shows the statistical correlation value between the Ordinal Clinical Progression Scale and the Dyspnea-12 Questionnaire. These two variables exhibit a statistical significance (p < 0.05) through their association measured as r = 0.521. Clinical progression severity shows a moderate positive correlation with dyspnea symptoms according to the test results. The findings demonstrate that when clinical COVID-19 progression increases according to the Ordinal Clinical Progression Scale, patients will report heightened dyspnea symptoms using the Dyspnea-12 Questionnaire. Clinical outcomes that result in severe patient deterioration lead to increased reports of respiratory distress from affected individuals.

**Table 3:** Intercorrelations between the study variables *: p<0.05, **: p<0.001 considered significant; correlation: Pearson Correlation

Variable	Ordinal Clinical Progression Scale	Dyspnea-12 Questionnaire
Ordinal Clinical Progression Scale	-	0.521^*^
Dyspnea-12 Questionnaire	0.521^*^	-

Table 4 presents a comparison of the Ordinal Clinical Progression Scale and Dyspnea-12 scores between those given oxygen and those not given oxygen. People treated with oxygen experienced higher scores for clinical progression (M = 3.67, SD = 1.56) than those who did not receive oxygen (M = 3.25, SD = 1.64), t (383) = 2.457, p = 0.01 and the effect size was small (Cohen’s d = 0.26). Dyspnea-12 Questionnaire scores were slightly higher in the group using oxygen therapy (M = 30.1, SD = 1.33) than in the group not using it (M = 29.9, SD = 1.49) and the difference was found to be significant, t(383) = 1.185, p = 0.024, but the effect size was extremely small (Cohen’s d = 0.14). The outcomes mean that individuals who used oxygen therapy are thought to have had slightly more severe symptoms and dyspnea.

**Table 4:** Comparison among variables (received oxygen therapy) M: Mean; SD: standard deviation; LL: lower limit; UL: upper limit; Cl: confidence interval; Independent t-test, *: p<0.05, **: p<0.001 considered significant

Variable	Yes (N=249)	No (N=136)	t	P	Cl 95%		Cohen’s D
	M±S.D	M±S.D			LL	UL	
Ordinal Clinical Progression Scale	3.67±1.56	3.25±1.64	2.457	0.01	.083	.750	0.26
Dyspnea-12 Questionnaire	30.1±1.33	29.9±1.49	1.185	0.024	-.116	.468	0.14

Table 5 shows how the paths and levels of dyspnea differed for patients treated in an ICU (N = 175) and those who were not (N = 210). Those treated in the ICU (mean = 3.36, SD = 1.64) scored lower on the Ordinal Clinical Progression Scale than those who were not (mean = 3.65, SD = 1.55) and the difference was highly significant (t = -1.790, p < 0.001). The confidence interval covered values from -0.614 to 0.029, showing that the true effect might be negative or quite close to zero and the effect size was not very strong (Cohen’s d = 0.18). On the Dyspnea-12 scale, the ICU patients (mean = 30.1, SD = 1.48) showed slightly higher scores than the non-ICU group (mean = 29.9, SD = 1.31) and this difference was considered statistically significant (t = 1.385, p = 0.001). The values in the confidence interval (-0.083, 0.477) led to a tiny possible effect that can be ignored, and the effect size went in the same direction (Cohen’s d = 0.14). Although there were differences between ICU and non-ICU patients, the small effect sizes suggest that those differences might not matter very much in practice.

**Table 5:** Comparison among variables (received ICU care) M: mean; SD: standard deviation; LL: lower limit; UL: upper limit; Cl: confidence interval; Independent t-test, *: p<0.05, **: p<0.001 considered significant

Variable	Yes (N=175)	No (N=210)	t	P	Cl 95%		Cohen’s D
	M±S.D	M±S.D			LL	UL	
Ordinal Clinical Progression Scale	3.36±1.64	3.65±1.55	-1.790	<0.001	-.614	.029	0.18
Dyspnea-12 Questionnaire	30.1±1.48	29.9±1.31	1.385	0.001	-.083	.477	0.14

Table 6 shows a one-way ANOVA analysis to compare COVID-19 severity groups against the Ordinal Clinical Progression Scale and Dyspnea-12 Questionnaire scores from patients with mild (no pneumonia) and moderate (pneumonia without oxygen) conditions, severe (oxygen requirement) and critical (ICU/mechanical ventilation) conditions. 

**Table 6:** Comparison of variables (severity of COVID-19) M: mean; S. D: standard deviation; F: ratio of variance between groups to within groups; η^2^: effect size; one-way ANOVA; **: p<0.01 considered significant

Variable	Mild-no pneumonia (N=144)	Moderate-pneumonia, no oxygen (N=72)	Severe-oxygen required (N=91)	Critical-ICU/mechanical ventilation (N=78)	p	F (3,381)	η^2^
	M±S.D	M±S.D	M±S.D	M±S.D			
Ordinal Clinical Progression Scale	3.75±1.69	3.53±1.49	3.32±1.59	3.32±1.49	<0.001	2.888	0.22
Dyspnea-12 Questionnaire	29.9±1.45	30.0±1.03	30.2±1.45	29.9±1.53	<0.001	0.586	0.46

The Ordinal Clinical Progression Scale revealed its highest mean score level among those with mild symptoms (M=3.75, SD=1.69), followed by moderate cases (M=3.53) and severe group (M=3.32) and critical patients (M=3.32). The analysis of variance revealed a significant relationship between COVID-19 groups according to F(3, 381) = 2.888, along with an effect size η² = 0.22 for a small to moderate impact.

The results from the Dyspnea-12 Questionnaire revealed minimal variations between severity levels, showing scores from 29.9 to 30.2 with an F value of 0.586 and η² = 0.46. The analysis indicates that COVID-19 severity level does not influence patient reports of dyspnea symptoms, while the effect size confirms this assessment. The clinical measures indicated minimal variability based on disease severity, yet patients at all disease levels reported similar levels of dyspnea symptoms.

Table 7 shows the difference in clinical status and the presence of dyspnea, categorized according to the length of time since the individual recovered from COVID-19, which is less than one month, 1 to 3 months, 4 to 6 months, and more than six months. The ANOVA did not discover significant differences in Ordinal Clinical Progression Scale scores between the four groups (F (3, 381) = 1.806, p = 0.49, η² = 0.02). Although recovery time was associated with a small rise in mean scores, from 3.31 (SD = 1.48) early on to 3.75 (SD = 1.76) in the long-term group, this effect was not significant and explained very little of the results. There was a statistically significant difference in Dyspnea-12 scores over the same periods (F (3, 381) = 2.907, p = 0.04, η² = 0.44). Participants who got better more than six months before reported the most breathlessness (M = 30.50, SD = 1.57), and those with COVID for less than a month had the least (M = 29.6, SD = 1.60). Even though the result was significant, the effect size found (eta-squared = 0.44) tells us that the explained variance is low enough that we should carefully consider its clinical impact. The examination of recovery time showed only small effects on the progression of illness, but it seems to be linked with ongoing breathing issues for those who recovered more than six months in the past.

**Table 7:** Comparison of variables (time since recovery) M: mean, S. D: standard deviation, F: ratio of variance between groups to within groups, η^2: ^effect size; One-way ANOVA; **: p<0.01 considered significant

Variable	Less than 1 month (N=80)	1-3 months (N=219)	4-6 months (N=74)	More than 6 months (N=12)	p	F (3,381)	η^2^
	M±S.D	M±S.D	M±S.D	M±S.D			
Ordinal Clinical Progression Scale	3.31±1.48	3.53±1.61	3.69±1.69	3.75±1.76	0.49	1.806	0.02
Dyspnea-12 Questionnaire	29.6±1.60	30.1±1.22	30.2±1.54	30.50±1.57	0.04	2.907	0.44

Table 8 shows results from multiple regression analysis showing relationships between the Ordinal Clinical Progression Scale and Dyspnea-12 Questionnaire score as an assessment of postoperative quality of recovery. The regression model estimation produces a constant value of 29.942, which represents the predicted Dyspnea-12 score under the Ordinal Clinical Progression Scale at zero. A highly notable association exists between these variables at p < 0.001.

**Table 8:** Multiple regression for postoperative quality of recovery scale Constant: Dyspnea-12 Questionnaire; B: coefficient; S.E: standard error; β: standardized coefficient; LL: lower limit; UL: upper limit; Cl: confidence interval; **: p<0.01 considered significant.

Variable	B	95% Cl		S.E	β	P
		LL	UL			
Constant	29.942	29.60	30.28	0.172	-	<0.001
Ordinal Clinical Progression Scale	0.224	.066	.109	0.445	0.521	<0.001

Each point increase in clinical progression score leads to a 0.224-point average increase in Dyspnea-12 scores according to the research findings using the Ordinal Clinical Progression Scale (B = 0.224, 95% CI: 0.066 to 0.445). The strength of the relationship between clinical progression severity and reported dyspnea symptoms is moderate to strong, based on the standardized coefficient value of β = 0.521. The relationship demonstrated strong statistical importance (p < 0.001) because worsening clinical progression causes patients to experience elevated postoperative respiratory distress symptoms. The research demonstrates how severe disease affects breathing recovery among COVID-19 patients.

**Figure 1:**
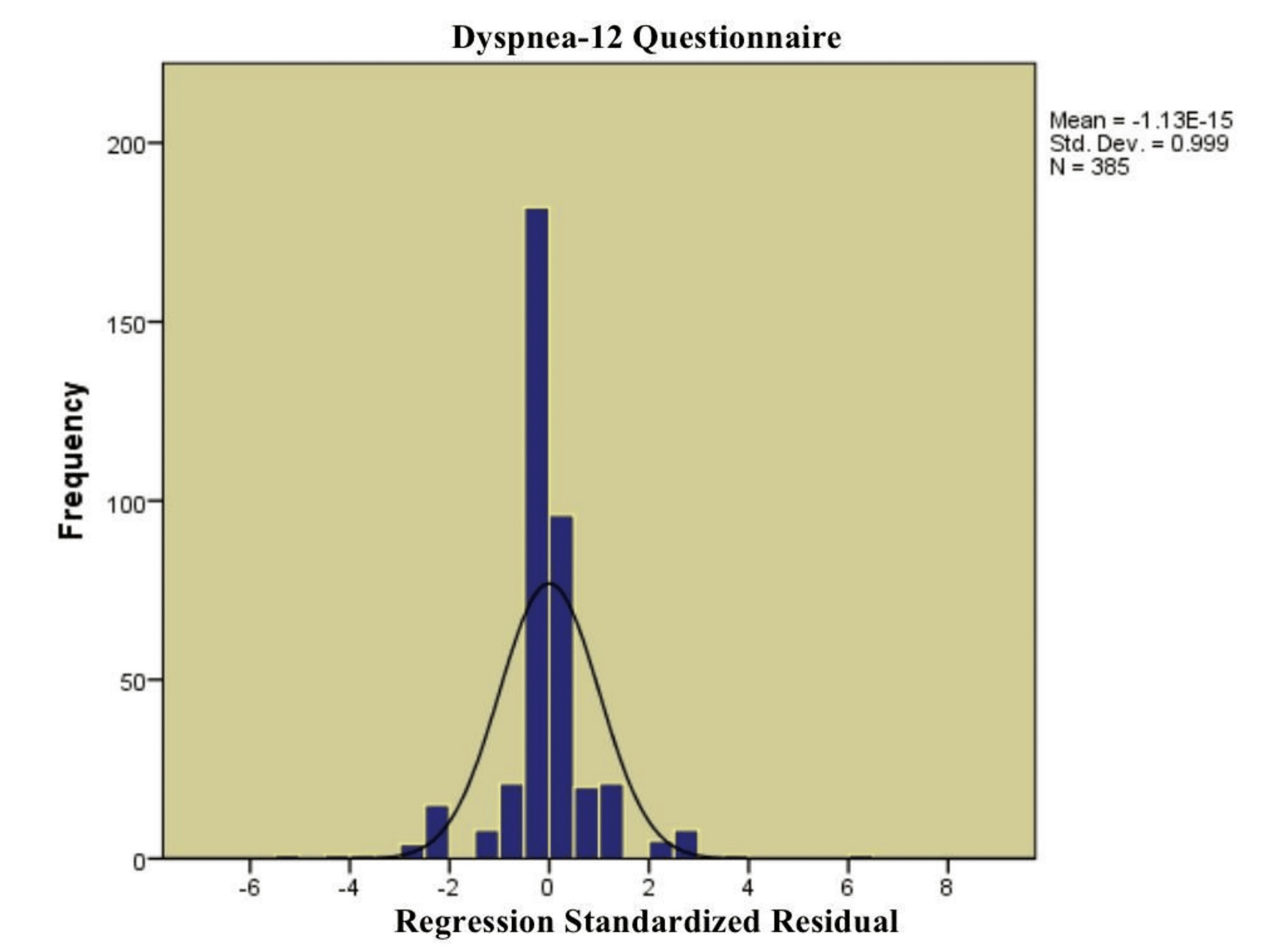
Histogram of linear regression of the Dyspnea-12 Questionnaire

Figure 1 reports a histogram of the standardized residuals of the linear regression analysis of Dyspnea-12 Questionnaire scores. A histogram visually tests the normality of residuals, which assumption is important in linear regression. The distribution looks approximately symmetrical and bell-shaped, which is an indication that a normal distribution of residuals is prevailing. The meaning of the standardized residuals is close to zero (−1.13), and their standard deviation is also within the range that is expected (0.95), further reinforcing the validity of the assumptions regarding the regression.

**Figure 2:**
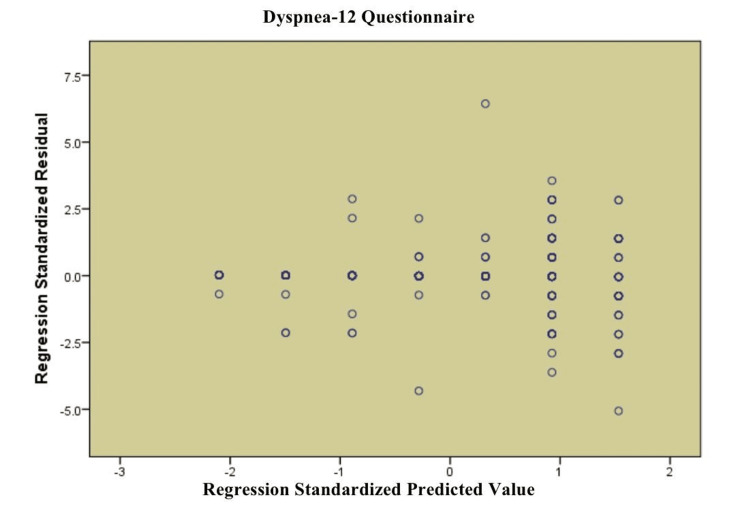
Scatterplot for linear regression of the Dyspnea-12 Questionnaire

Figure 2 shows a scatter plot depicting the standardized residuals vs. the standardized predicted values for the Dyspnea-12 Questionnaire regression model. This plot is used to test the assumption of homoscedasticity that the residuals should demonstrate a constant variance over the predicted value levels. According to the scatterplot, the residuals are randomly placed around the horizontal axis, and there is no definite pattern, which means that the assumption of homoscedasticity is reasonably fulfilled. More importantly, with no systematic structure lacking, it is indicative that the model does not have significant specification errors.

Table 9 shows demographic statistics with three variables of interest that include history of chronic illness and COVID-19 disease severity, and recovery duration. The analysis demonstrated that having a chronic illness creates a strong correlation (p < 0.001, χ² = 53.4) with severe COVID-19 manifestations in patients. According to the data analysis, individuals with hypertension (n=68) needed a combination of urgent severe oxygen therapy and treatment in intensive care units at rates of 29 and 15, respectively. The same pattern emerged for diabetes, asthma, and COPD patients since numerous cases of these chronic diseases evolved into severe or critical conditions. A significant relationship exists between the duration since recovery and having a chronic illness (p = 0.05, χ² = 14.02). Patients with asthma and COPD needed extensive time for recovery since medical experts observed that multiple persons remained in a recuperative state for three to six months. The study indicates that persistent diseases substantially influence COVID-19 clinical presentation and the duration of recovery needs.

**Table 9:** Descriptive statistics of demographic variables (history of chronic illness, severity of COVID-19, and time since recovery) f: frequency; %: percentage; p: level of significance; p-values calculated using the chi-square test; the significance level is set at p < 0.05

Variables	f		Severity of COVID-19			p	x^2^			Time since recovery		p	x^2^
		Mild-no pneumonia	Moderate pneumonia, no oxygen	Severe-oxygen required	Critical-ICU/mechanical ventilation			Less than 1 month	1-3 months	4-6 months	More than 6 months		
History of Chronic Illness	-	-	-	-		<0.001	53.4	-	-	-		0.05	14.02
Hypertension	68	11	13	29	15	-	-	18	40	10	0	-	-
Diabetes	61	26	11	11	13	-	-	14	34	11	2	-	-
Asthma	73	35	18	9	11	-	-	11	43	16	3	-	-
COPD	72	36	12	14	10	-	-	18	38	14	2	-	-
Heart disease	64	31	7	12	14	-	-	15	31	15	3	-	-
Kidney disease	47	5	11	16	15	-	-	4	33	8	2	-	-

Table 10 examines the correlation between hospitalization duration, COVID-19 severity, and time since recovery. There is a very significant correlation between hospitalization duration and COVID-19 severity (p < 0.001, χ² = 42.30), with increased hospitalization duration corresponding to increased severity. For instance, those who were admitted for over two weeks had the most frequent severe symptoms, where 46 required oxygen and 48 needed ICU treatment. On the other hand, those admitted for under a week had predominantly mild to moderate symptoms. In addition, the length of hospitalization was also independently related to recovery time (p < 0.001, χ² = 10.67). Those who were hospitalized for more than two weeks were also more likely to have more extended recovery periods, with most still recovering after more than six months. This highlights how more extended hospital stays are associated with greater severity and more extended recovery periods.

**Table 10:** Descriptive statistics of demographic variables (duration of hospitalization, severity of COVID-19, and time since recovery) f: frequency; %: percentage; p: level of significance; p-values calculated using the chi-square test; the significance level is set at p < 0.05

Variables	f		Severity of COVID-19				p	x^2^			Time since recovery	
		Mild-no pneumonia	Moderate pneumonia, no oxygen		Severe-oxygen required	Critical-ICU/mechanical ventilation			Less than one month	1-3 months	4-6 months	More than six months
Duration of Hospitalization	-	-	-		-	-	<0.001	42.30	-	-	-	-
Less than one week	105	43	28		24	10	-	-	24	54	20	7
1-2 weeks	122	65	16		21	20	-	-	28	69	24	1
More than 2 weeks	158	36	28		46	48	-	-	28	96	30	4

## Discussion

The objective of this research was to investigate the correlation between the severity of COVID-19 infection and the long-standing presence of dyspnea among adults who recovered from the disease. Our results are consistent with earlier research indicating that severe COVID-19 progression is linked to persistent respiratory complications. As the pulmonary limitations experienced by survivors, we saw a moderate positive correlation between clinical progression severity and perceived dyspnea, implying that severe illness is responsible for long-term breathing problems, even several months after recovery [18].

Our results are in line with research indicating that patients with more severe COVID-19, as measured by greater clinical progression scores, are at greater risk of needing oxygen therapy. Like previous research, dyspnea and respiratory distress were the most important factors linked to the need for oxygen, demonstrating their utility as markers of illness severity [19].

Consistent with past studies, our analysis showed that dyspnea was a major predictor of severe COVID-19 and ICU admission. Comorbid conditions such as COPD and cardiovascular disease, found in the literature, also increase the risk of severe illness [20]. The findings of our research confirm earlier findings that mild cases of COVID-19 can escalate to severe phases. Certain factors are responsible for this escalation, and thus, monitoring at an early stage is essential in vulnerable patients [21]. Our research established that dyspnea was comparatively stable across varying levels of severity, which is unlike other studies where dyspnea was recognized as a robust predictor of severe COVID-19. The variability may result from differing populations or methodological differences between studies [22].

The present study revealed that the severity of dyspnea increased with longer recovery time. This is consistent with previous research indicating that poor muscle strength after COVID is associated with increased dyspnea and poor quality of life, highlighting the importance of physical rehabilitation in long-term recovery [23]. Results of the present study identified that increased disease severity correlates with increased dyspnea scores during recovery. This is consistent with previous evidence demonstrating that dyspnea independently predicts longer recovery times from COVID-19, underscoring the necessity for specific respiratory monitoring and support [24].

Chronic comorbidities, including hypertension, diabetes, and chronic pulmonary illnesses, were significantly linked with increased severity and extended recovery in COVID-19 patients. These results align with earlier studies, which point out that underlying conditions play a significant role in poorer outcomes and more extended recovery periods [25,26].

In our research, a high correlation was found between more extended hospital stays and greater severity of COVID-19, as well as more extended recovery periods. This is consistent with other studies, which found higher age, increased neutrophil count, CRP, and D-dimer levels as predictors of more extended hospital stay as well as increased risk of complications [27]. Our results concur with previous literature, revealing that more extended hospital stays are significantly linked to severe COVID-19 symptoms and more extended recovery periods, as has been found in prior research where more extended stays have been shown to correlate with greater disease severity and more extended recovery periods [28].

Limitations and future directions

There are some obstacles to this research. First, since this is a cross-sectional study, it cannot be concluded that COVID-19 severity directly leads to more cases of dyspnea. Also, when people depend on their memories or descriptions, their answers may not be accurate for symptoms like length and intensity. The third point is that the research focused on a particular group of people, which might limit how widely the results can be applied. Other underlying conditions, such as pulmonary or mental health disorders, were not fully considered, making it hard to interpret the observed results. Even though we noticed small effects, we made it clear that our findings, which are significant, are not likely to represent a big change in how severe dyspnea is experienced. Also, little changes may make a difference to patients with mild symptoms because they can help a lot with their recovery.

Future studies should use longitudinal designs to further define causality and follow post-COVID-19 symptoms over time. Using a larger and more diverse population from multiple regions would increase generalizability. Objective measures, including pulmonary function tests or imaging, in addition to patient self-reporting, should be included in studies to give a better picture of dyspnea. In addition, investigating the function of rehabilitation, psychological determinants, and other comorbidities could assist in designing targeted interventions for patients with enduring symptoms.

## Conclusions

This research illustrates a strong correlation between the severity of COVID-19 infection and the persistence of dyspnea among recovered adult patients. Patients who have had moderate to severe COVID-19 are likely to experience continuing respiratory symptoms after recovery. The results emphasize post-recovery monitoring and care, especially for severe initial infections, and highlight the need for an integrated care model to address the long-term consequences of COVID-19.

## Appendices

**Table 11:** Demographic information questionnaire

Items	Responses
Age	-
Gender	-
Marital Status	-
Education Level	-
Employment Status	-
Smoking Status	-
History of Chronic Illness	-
COVID-19 Severity	-
Hospitalized for COVID-19	-
If Yes: Duration of Hospitalization	-
Received Oxygen Therapy During Infection?	-
Received ICU Care?	-
Time Since Recovery	-
